# A thioredoxin reductase inhibitor ethaselen induces growth inhibition and apoptosis in gastric cancer

**DOI:** 10.7150/jca.40744

**Published:** 2020-03-04

**Authors:** Wang Wu, Zhiying Yang, Xing Xiao, Tailai An, Bo Li, Jun Ouyang, Huafu Li, Chunming Wang, Yawei Zhang, Haiyong Zhang, Yulong He, Changhua Zhang

**Affiliations:** 1 Center for Digestive Disease, the Seventh Affiliated Hospital of Sun Yat-sen University, Shenzhen, Guangdong, 518107, China; 2 Department of Gastrointestinal Surgery, the First Affiliated Hospital of Sun Yat-sen University, Guangzhou, Guangdong 510080, China

## Abstract

Gastric cancer (GC) is the third leading cause of cancer deaths worldwide. Conventional chemotherapy has been proven useful to only a portion of the patients. Previous developed targeted drugs are more effective and tolerable than conventional drugs. Thus the development of novel drugs targeting markers is an urgent task and the main direction for future research. Ethaselen, an inhibitor of thioredoxin reductase (TrxR), has been considered an important anticancer target drug. Previous studies show that it is effective on treating many kinds of cancers. In this paper, we examined that ethaselen effectively inhibited the growth of gastric cancer cells and promoted apoptosis. Organoids were cultured from patient-derived cells in a three-dimension form which are widely used in cancer research to help us understand cancer cells behavior at the sub-organ level and develop novel drugs. We established a drug testing and screening system using GC-derived organoids by recapitulating tumor microenvironment. We confirmed that the TrxR-targeting ethaselen could be a novel and effective drug for gastric cancer treatment.

## Introduction

Many studies have demonstrated that the expression quantity of thioredoxin (Trx) in cancer cells is significantly higher than that in normal cells. Trx has been proven to be closely associated with cell proliferation and apoptosis for a long time. Thioredoxin reductase (TrxR) is a major antioxidant that plays an irreplaceable role in maintaining the redox equilibrium within cells. TrxR reduces a target protein by converting its disulfide bond to two-SH groups, with its two-SH groups being oxidized to form a disulfide bond. TrxR is a selenoprotein that reduces the oxidoreductase thioredoxin in a NADPH-dependent manner. So far, two forms of mammalian TrxRs are identified as TrxR1 and TrxR2, which are located in the cytoplasm and mitochondria respectively^1^,^2^. Despite in cells, two isoforms are located, TrxR1 and TrxR2 have a similar structure and catalytic function to each other. Overexpression of TrxR1 has been detected in many human tumors^3^. Reactive oxygen species (ROS) are normal byproducts of numerous cellular processes, such as mitochondrial metabolism and protein folding^4^. At a low concentration, ROS acts as signaling molecules to activate proliferation and survival pathways^5^. A modest increase of ROS concentration damages DNA and induces mutations^6^,^7^. An higher ROS concentration causes cell senescene or death^8^. In order to eliminate oxidative stress, cells produce antioxidants to convert ROS into harmless molecules. Comparing to normal cells, cancer cells usually possess higher level of ROS and antioxidant activities in an uncontrolled way^9^. Consequently, cancer cells are unable to cope with additional oxidative stresses^10^. Therefore, increasing the ROS production by thioredoxin reductase is a theoretically and practically feasible strategy for cancer treatment.

As an inhibitor of thioredoxin reductase, Ethaselen(BBSKE) is known to selectively kill cancer cells while it spares normal cells. Many studies have demonstrated that BBSKE can interact with TrxR1 both *in vitro* and *in vivo*^11^,^12^. By inhibiting TrxR1 activity and increasing intracellular ROS, BBSKE induces a lethal endoplasmic reticulum stress and mitochondrial dysfunction in human gastric cancer cells^12^. *In vivo*, BBSKE remarkably reduces the TrxR1 activity and fortifies cancer cell stress burden^13^. Additionally, TrxR1 highly expressed in gastric cancer cells and human gastric cancer tissues. Targeting TrxR1 by drugs may be a feasible mechanism in treating cancers^14^.

Organoids are three-dimensional *in vitro* models that can be utilized to study the biology of human cancers along with their interactions with the micro-environment^15^,^16^,^17^. Although the traditional cancer cell lines are valuable in the investigation of fundamental cancer research mechanisms, these models have the significant disadvantage and little resemblance to the intended patient tumor^18^,^19^,^20^,^21^. Therefore, organoids are widely used as pre-clinical models given their unique advantages: highly consistency with the original tumor regarding biological behavior and heterogeneity; stability of the genome after repeated passagings; short culturing period and low costs^22^,^23^,^24^,^25^. And the treatment of patient-derived organoids will ultimately test their usefulness to predict individual therapy response and patient outcome^25^. Considering all these advantages, we cultured organoids from gastrointestinal tumor patients and tested the efficiency of BBSKE in treating gastric cancer.

## Materials and methods

### Cell culture and reagents

BBSKE was synthesized and provided by the Department of Chem-Biology, School of Pharmaceutical Sciences, Peking University, China. Human gastric cancer cell lines SGC-7901, BGC-823, MGC-803 and HGC-27 were purchased from the Institute of Biochemistry and Cell Biology, Chinese Academy of Sciences. The cells were routinely cultured in RPMI 1640 and DMEM/F12 medium (Gibco, Eggenstein, Germany) containing 10% heat-inactivated fetal bovine serum (Gibco, Eggenstein, Germany), 100 units/mL penicillin, and 100 μg/mL streptomycin in a humidified cell incubator with an atmosphere of 5% CO_2_ at 37℃. The metrigel was purchased from USA. Antibodies including anti-Bcl-2, anti-caspase-3, anti β-actin, goat anti-mouse IgG-HRP and goat anti-rabbit IgG-HRP were purchased from Santa Cruz Biotechnology (Santa Cruz, CA). FITC Annexin V apoptosis Detection Kit I and Propidium Iodide (PI) were purchased from Transgen biotech Beijing.

### Cell viability assay

Cells were seeded into 96-well plates at a density of 8000 per well. BBSKE was dissolved in DMSO and diluted with DMEM medium to final concentrations of 2.5, 5, 10, 20, 40 μM. The tumor cells were incubated with BBSKE in a CO_2_ incubator at 37℃ for 24h before the CCK8 assay. The absorbance was then measured at 450 nm using a microplate reader.

### Determination of TrxR activity

The TrxR activity of different cell lines, normal gastric tissues and tumor tissues were measured by elisa. All standards and samples were added in duplicate to the Microelisa Stripplate. The wells for standard were set and the wells for sample were tested. The color of the liquid in the wells should change from blue to yellow as expected. If the color is green or the color does not change uniformly, we gently tapped the plate to make sure of thorough mixing. Data of the Optical Density (O.D.) at 450 nm were read out using a microtiter plate reader within 5 minutes.

### Cell apoptosis analysis

Apoptosis was evaluated by Annexin V-FITC/PI staining assay. HGC-27, SGC-7901, BGC-823 and MGC-803 cell lines were seeded on 60-mm dishes and incubated for 24h and then treated with BBSKE (10, 20 or 40 μM) for 24h. The samples were analyzed using Flex Flow Cytometer (Beckman Coulter, Brea, CA, USA). Annexin V (+)/PI (-) cells were identified at the early stages of apoptosis detection and Annexin V (+)/PI (+) cells were identified at the late stages.

### Western blotting analysis

Cells were homogenized in protein lysate buffer and debris was removed by centrifugation at 12,000 g for 10 mins at 4 °C. The protein concentrations of the samples were determined using the BCA assay. After the addition of sample loading buffer, the samples were electrophoresed and then transferred to poly-vinylidene difluoride transfer membranes. After being blocked for 1 hour at room temperature with 5% BSA, the samples were then incubated with specific primary antibody overnight at 4 °C. After being washed three times with TBST, the samples were incubated with horseradish peroxidase-conjugated secondary antibody for 1 hour. Then the immunoreactive bands were visualized using chemidoc imaging system. Densities of the immunoreactive bands were analyzed using quantity one software.

### Patients' information and tissue samples

This study was approved by the Institutional Research Human Ethical Committee of the Sun Yat-Sen University for the use of clinical specimens and informed consents were obtained from all the patients. A total of 10 gastric cancer patients that were clinically diagnosed at the Seventh Affiliated Hospital of Sun Yat-sen University during the period of 2018 to 2019 were included. Gastric cancer tissues and the matched tumor-adjacent morphologically normal gastric tissues were frozen and stored in liquid nitrogen for further use.

### Organoids culture

Gastric cancer tissues were immediately obtained from the resected specimens. Using sterile forceps, transfer the specimen to a sterile tissue culture dish and mince the remaining specimen into small fragments (1 mm^3^ or less). Transfer remaining tissue to 5 mL protein LoBind tube containing the 3 mL Human Digestion Medium supplemented with DNAse and Y-27632. Place the tube in rotating incubator set to 37ºC with rapid rotation (35 rpm) for an initial digestion of 15 minutes. Dilute the digest with 20 mL DPBS without Ca^2+^ and Mg^2+^ and filter the cells through a 70-mm filter. The cells were centrifuged at 1200 rpm for 5 minutes and embedded into matrigel supplemented with gastric growth medium. Matrigel was used to suspend organoids. After being transported in iced media, the materials were washed with antibiotic-rich washing liquid and mechanically or enzymatically processed to obtain cell clusters. Plate 50 µL matrigel domes into a pre-warmed 24 well plates and placed in 37℃ tissue culture incubator until matrigel solidifies then cultured in freshly generated human gastric organoid media. Organoids were observed and images were captured using a laser scanning confocal microscope (LSM710, Carl Zeiss, Germany).

### Statistical analysis

All experiments were assayed in triplicate. Data are expressed as means ± SEM. All statistical analyses were performed using Graph Pad Pro. Prism 5.0(GraphPad, SanDiego, CA). The differences among sets of data were analyzed via Student's t-test and two-way ANOVA. It was considered statistically significant when P was less than 0.05 (* p < 0.05, ** p < 0.01, *** p < 0.001).

## Results

### BBSKE inhibits the growth of gastric cancer cell lines

**Figure 1:**
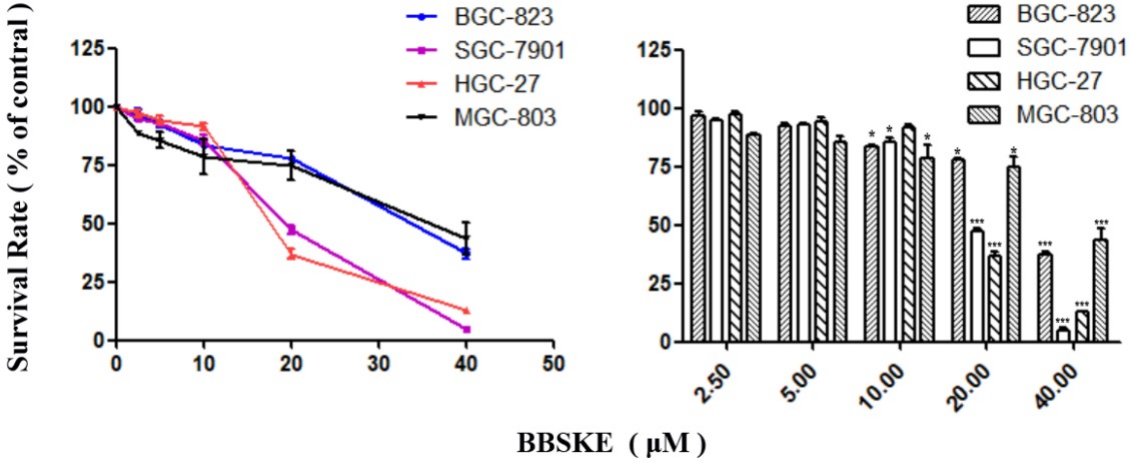
Effects of BBSKE on cellular proliferation by CCK8 assay. The effects of BBSKE on the proliferation of human gastric cancer cell line. BGC-823, SGC-7901, HGC-27 and MGC-803 cells were incubated with increasing doses of BBSKE (0-40 μM) for 24 h. Data represent similar results from three independent experiments.

After being treated with BBSKE for 24 hours, exponentially growing cells were analyzed by CCK-8 assay. The growth of gastric cancer cells were inhibited dependently (Fig. 1). At the concentration of 20 μM, the inhibitory effects of BBSKE on SGC-7901 and HGC-27 were higher than those on BGC-823 and MGC-803. And all the cells were suppressed significanly by 40 μM BBSKE, with an inhibition rate of over 50%.

### BBSKE increases apoptosis on gastric cancer cell lines

**Figure 2:**
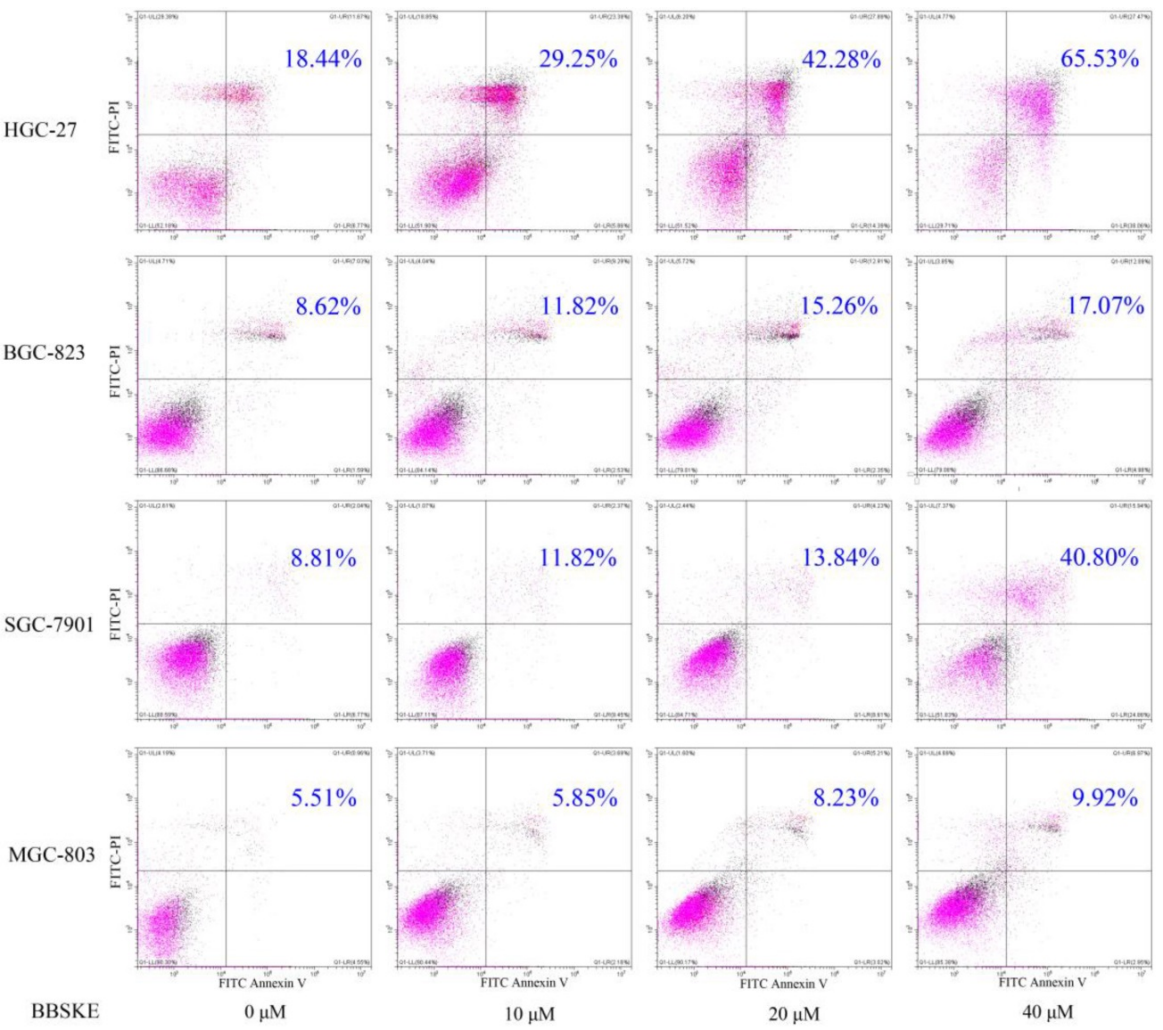
Cells were assayed by Annexin V-FITC/PI staining after 24h treatment with various dosages of BBSKE. Drug dosages and apoptotic proportions were labeled in each figure. BBSKE induces apoptosis in human gastric cancer cells BGC-823, SGC-7901, HGC-27 and MGC-803. Percentage of cell apoptosis was determined by Annexin-V/PI staining using flow cytometry.

To examine the apoptosis effect of BBSKE, cells were treated with various concentrations of BBSKE, and then were assessed by Annexin V/ propidium iodide (PI) double staining assay. All of four gastric cancer cell lines showed a concentration dependent apoptosis after 24 hours treatment with BBSKE (Fig. 2). The results were consistent with the results of cytotoxicity by CCK-8 assay.

### BBSKE depresses the protein expressions of bcl-2 and caspase-3

**Figure 3:**
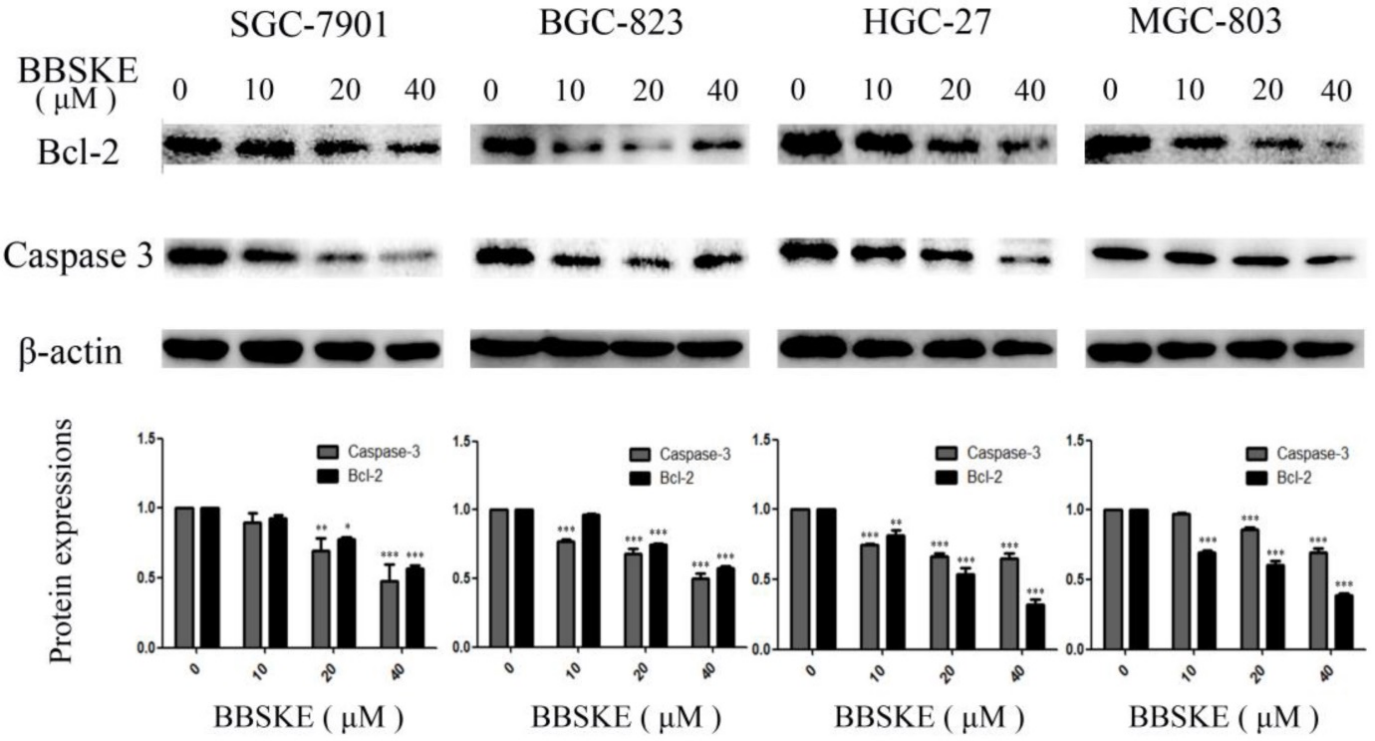
The expressions of the apoptosis associated protein Bcl-2 and caspase-3. The gastric cells were treated with different concentrations of BBSKE for 24 h and then assayed by western blot. β-actin was used as a lane loading control.

Two proteins that involved in apoptosis, such as Bcl-2 and Caspase-3 were quantified by western blotting. After being treated with BBSKE for 24 hours, the expressions of Bcl-2 and Caspase-3 in the studied cell lines were significantly down-regulated (Fig. 3).

### The concentration of TrxR is decreased by BBSKE in gastric cancer cells

**Figure 4:**
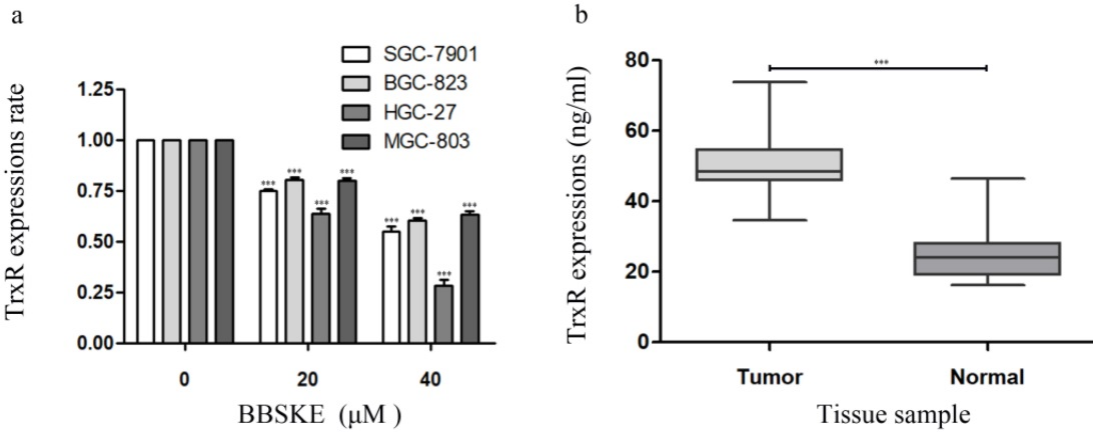
The concentration of TrxR was decreased by BBSKE in gastric cancer cells. The data above was verified by Elisa assay. a: The expression levels of TrxR in the four cell lines were significantly different at different concentrations. The influences of BBSKE on SGC-7901 and HGC-27 are significantly higher than that of BGC-823 and MGC-803. b: And compared with normal and tumor tissues, the TrxR expression in tumor tissues was also significantly higher than that in normal tissues.

We investigated the relationship between TrxR activity and cell viability after BBSKE was added to each cultured cell line. The results showed that, the activity of TrxR was downregulated with the increase of cell inhibition rate after 24 hours (Fig. 4a). Furthermore, the TrxR activity of the gastric cancer tissues and normal tissues was investigated, the results of which were listed in figure 4b. And the level of TrxR in cancer tissues was significantly higher than that in normal tissues.

### BBSKE inhibits the growth of cancer tissue-derived organoids

**Figure 5:**
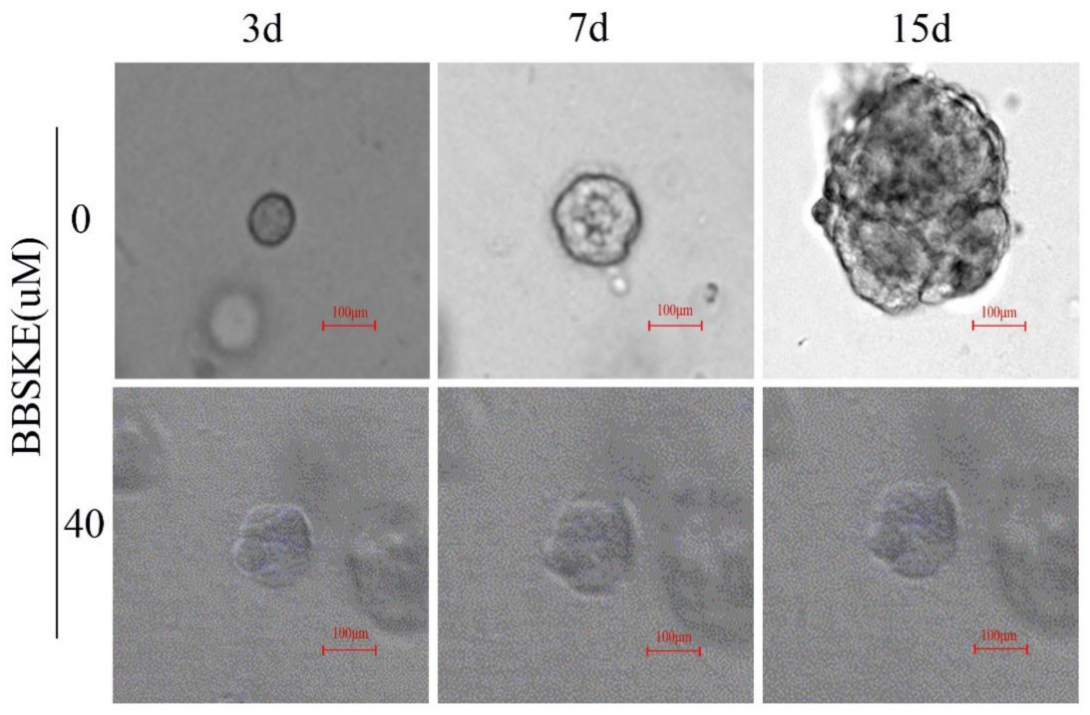
We cultured 7 cases organoids of cancer tissues from 5 patients with gastric cancer, and observed the effect on their growth at 40 μM BBSKE, compared with the control group (0µM BBSKE) by confocal microscope.

All the results show that the BBSKE has a negative effect on the four gastric cancer cells above. In this part, we observe the similar results in the model of organoids. After treatment with BBSKE, the patient-derived organoids stop growing while the shape of control was much larger after 15 days culture (Fig. 5).

## Discussion

The development of novel treatment regimens is the challenge of translating scientific knowledge from bench to bedside, which is mainly due to the fact that many cancer models only poorly recapitulate the patient's tumor, and as a consequence, many drugs that perform well in cancer models ultimately fail in clinical trials^25^. Common used human cancer models include cancer cell lines and primary patient-derived tumor xenografts (PDTXs). Conventionally, it is via the cultured cell lines that the cytotoxic effect of a drug is tested^18^,^19^. Cancer cell lines are derived from primary patient material and have contributed tremendously to cancer research. However, they have several drawbacks. For instance, their generation from primary patient material is very inefficient and involves extensive adaptation and selection to *in vitro* 2D culture conditions. As only rare clones are able to expand and can be maintained over many passages, the derived cell lines may have undergone substantial genetic changes and no longer recapitulate the genetic heterogeneity of the original tumors. Other limitations of cell lines include the absence of normal tissue-derived control cell lines as reference and the lack of stromal compartments. Although animal cancer models provide important insights into the basics of cancer, their generation is time consuming, and it is argued that these models often do not faithfully recapitulate pathogenic processes in patients, the histological complexity and genetic heterogeneity of human cancers are typically not reflected in genetically engineered mouse models of cancer. And PDTXs may undergo mouse-specific tumor evolution^27^,^28^.

Fortunately, organoids can simulate nutrient and oxygen supplies for 3D complex organ systems *in vivo*, as an incubator based on the strategy to develop organoids, making great strides toward the realization of regenerative therapy^29^. The organoids help explore how organs form and change in desease and can much better recapitulate the original tumor than cell lines and may be superior models to develop and test new anti-cancer drugs. In our study, we use a three-dimension system organoid model to detect BBSKE cytotoxic effect on gastric cancer (Fig. 5). High-throughput drug screening method using tumor tissue-derived organoids has just gained popularity among the academic community.

We demonstrated that for different times, the addition of BBSKE into the medium inhibited gastric cancer cells growth and the cytotoxic effect in a dose-dependent manner (Fig. 1). For example, at concentrations of 20 μM and 40 μM, cell inhibition was evident. Next, the apoptosis of the gastric cells induced by BBSKE was further measured. Apoptosis was detected by the method of Annexin V/(PI) double staining. Apoptosis occurred to all the gastric cancer cell lines in a concentration-dependent way 24 hours after BBSKE was added into the medium (Fig. 2). Similar results were observed in apoptosis detection via the determination of caspase-3 activity. Western blotting showed that BBSKE treatment could also dose-dependently inhibit the expression of cycle-related proteins (bcl-2) in these gastric cancer cells (Fig. 3).

The thioredoxin system has a novel target for treating cancer^6^, due to these following evidences: both Trx1 and TrxR1 excessively express in a variety of human cancers; overexpressions of Trx1 and Trx2 are significantly associated with more rapid tumor growth, drug resistance and poorer prognosis^1^,^2^,^3^. To further confirm how BBSKE affects Trx1 activity in gastric cancer cells, a few common gastric cell lines were used in our study. With the increasing of BBSKE, the activity of Trx1 significantly decreased (Fig. 4a). Therefore, the inhibitory effect of BBSKE on Trx1 was dose-dependent. Balanced by TrxR modulation, Trx1 is positive within proliferated viable cells and negative within apoptotic ones. But few reports on cellular TrxR exist. Using CCK8 assay, we quantified the TxrR activities in a few common used gastric cell lines. Furthermore, the expressions of TrxR in normal and gastric cancer tissues were determined (Fig. 4b).

BBSKE, a novel organic selenium compound, is chosen as a candidate for anticancer agent because of its lower toxic effect^12^,^13^,^30^,^31^,^32^. In this work, BBSKE significantly induced TrxR inactivation in the four cancer cell lines paralleled with growth inhibition. A good linear correlation (Fig. 3) between TrxR activity and cell viability was found in each cell line and this high pertinency confirmed that TrxR activity indeed contributed to cancer cell viability. In addition, comparing the gastric tumor tissue and normal tissue, we found that the concentration of TrxR in gastric cancer is higher.

Considering that TrxR is involved in tumor cells^33^, apoptosis resistance and BBSKE can effectively inhibit TrxR activity, we demonstrated that apoptosis was induced in all investigated gastric cancer cell lines after high doses of BBSKE treatment. It revealed that for the four gastric cell lines investigated in our study, the concentrations at which apoptosis was induced were different, which implied different sensitivity to BBSKE. And we calculated the IC50 (µM) of different cell lines, which was 14.27, 7.93, 17.02, and 10.59 for SGC-7901, HGC-27, BGC-823, and MGC-803 respectively (data is not given). A fundamental notion of apoptosis is that apoptotic signaling cascades usually stem from mitochondria and converge at the production of Bcl-2, while the capases are the ultimate executioners of cell death. For the four different investigated cell lines, BBSKE down-regulated the Bcl-2 and Caspase-3 expressions. These results of our study suggested the apoptosis induced by high-dose BBSKE was mediated by the Bcl-2 pathway.

In conclusion, our studies provide experimental evidence that by inactivating TrxR, BBSKE has an inhibitory effect on SGC-7901, HGC-27, BGC-823, MGC-803 cells. The proved inhibitory effect of BBSKE on cancer cells could help to be a potential agent against gastric cancer. More *in vitro* and *in vivo* studies should be warranted to establish the role of BBSKE as a therapeutic agent against gastric cancer.
